# Magnetic Resonance Image Denoising Algorithm Based on Cartoon, Texture, and Residual Parts

**DOI:** 10.1155/2020/1405647

**Published:** 2020-04-01

**Authors:** Yanqiu Zeng, Baocan Zhang, Wei Zhao, Shixiao Xiao, Guokai Zhang, Haiping Ren, Wenbing Zhao, Yonghong Peng, Yutian Xiao, Yiwen Lu, Yongshuo Zong, Yimin Ding

**Affiliations:** ^1^Chengyi University College, Jimei University, Xiamen, China; ^2^School of Software Engineering, Tongji University, Shanghai, China; ^3^Jiangxi University of Science and Technology, Nanchang, China; ^4^Department of Electrical Engineering and Computer Science, Cleveland State University, Cleveland, OH 44115, USA; ^5^Faculty of Computer Science, University of Sunderland, Sunderland, UK; ^6^School of Informatics, Xiamen University, Xiamen, China; ^7^Department of Computer Science, Tongji University, Shanghai, China; ^8^College of Electronics and Information Engineering, Tongji University, Shanghai, China

## Abstract

Magnetic resonance (MR) images are often contaminated by Gaussian noise, an electronic noise caused by the random thermal motion of electronic components, which reduces the quality and reliability of the images. This paper puts forward a hybrid denoising algorithm for MR images based on two sparsely represented morphological components and one residual part. To begin with, decompose a noisy MR image into the cartoon, texture, and residual parts by MCA, and then each part is denoised by using Wiener filter, wavelet hard threshold, and wavelet soft threshold, respectively. Finally, stack up all the denoised subimages to obtain the denoised MR image. The experimental results show that the proposed method has significantly better performance in terms of mean square error and peak signal-to-noise ratio than each method alone.

## 1. Introduction

Magnetic resonance imaging (MRI) is one of the advanced imageological examination methods for modern medicine. MRI uses powerful magnets and computer-generated radio waves instead of injected contrast agents to create multidimensional images of human organs and tissues. It does not damage the body with ionizing radiation, so it is safer than emission computed tomography (ECT). For this reason, MRI is frequently used for imaging tests of the brain and spinal cord. However, compared with computed tomography (CT), MR image has a lower spatial resolution, longer scan time, and more artifacts. The longer the scanning time, the greater the thermal noise (a kind of Gaussian noise). Besides, medical images are always polluted by various noises during collection, transmission, and storage. The magnitude of MRI data in the presence of noise generally follows a Rician distribution if acquired with single-coil systems [1]. Also, the Gaussian distribution can approximate Rician noise in high SNR (signal-to-noise ratio) regions [2]. Quite often, noise affecting the pixels in an image is Gaussian in nature and uniformly deters information pixels in the image [3]. MR image denoising, as an essential preprocessing step for MRI data processing, has been a hot topic in the related area.

Many scholars and researchers have performed much work on image denoising. Various image denoising methods can be broadly classified as five categories: spatial domain filtering, transform domain filtering, methods in other domains, sparse representation and dictionary learning methods, and hybrid methods [3].

The spatial domain filtering can be further divided into linear (such as Wiener filters) and nonlinear filters (such as median filters) [4]. Wiener filter, a denoising method used when the noise is a stationary random process, minimizes the mean square error between the output signal and the desired noise-free signal. It has a wide range of applications, regardless of whether the stationary random process is continuous or discrete, scalar or vector. Jingdong Chen et al. studied the quantitative performance behavior of the Wiener filter in the context of noise reduction in 2006 [5]. A new filtering method based on the neutrosophic set approach of the Wiener filter was proposed for MRI denoising in 2013 [6].

Transform domain filtering includes many classic methods, such as Fourier transform, wavelet transform, threshold function, curvelet, and contourlet. Wavelet transform uses a series of wavelets with different scales to decompose the original image to get the coefficients of different wavelets. In general, for a noisy image, the wavelet transform will decompose most of the noises and effective signals into coefficients with small and big moduli, respectively. So, it is easy to remove lots of the noises by removing the low-frequency parts. Wavelet thresholding is a powerful denoising method based on the wavelet transform. Donoho and Johnstone gave two kinds of thresholding functions: hard thresholding and soft thresholding [7, 8]. The deficiency of hard thresholding is its discontinuity, while the drawback of soft thresholding is that it causes constant deviation [9]. Fei Xiao and Yungang Zhang explored the properties of several representative thresholding techniques in wavelets denoising in 2011 [10]. Zhang et al. proposed an improved threshold function to overcome the drawbacks of hard thresholding and soft thresholding functions in 2019 [9]. Wavelet transform has been widely applied in image processing [11, 12].

**Figure 1:**
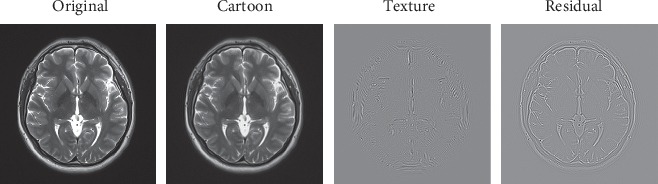
An original MRI and its decomposition based on MCA.

Hybrid methods are popular since different denoising methods have different advantages. Here, we focus on the application of morphological component analysis (MCA) and wavelet thresholding. MCA is a signal separation method proposed by Starck et al. in 2005 [13], which combines the advantages of sparse representation and variational method. When the features contained in an image present different morphological aspects, we can separate multiple components with different shapes from the image (as shown in Figure 1). The morphological component analysis assumes that each morphological component can find a dictionary for sparse representation, and the dictionaries for different morphological components are independent. The authors in [14] proposed an image denoising method based on morphological component analysis (MCA) to remove the rain component successfully in 2013. Naimi et al. proposed a denoising approach basing on dual-tree complex wavelet and shrinkage with the Wiener filter technique in 2014 [15]. Deng and Liu proposed an improved image denoising method based on MCA and median filter to resist mixed noises in 2015 [16]. Cheng and Liu used APBT and BM3D to denoise texture and structure part, respectively [17]. MCA has been widely used in medical image processing [18].

In this paper, we propose a method to remove Gaussian noise in MR brain images based on image decomposition by MCA. Wiener filter (classical spatial domain filtering), wavelet hard threshold, and wavelet soft threshold (classical transform domain filtering) are employed to remove noise in the cartoon, texture, and residual parts, respectively. The experimental results show that the proposed method achieves better noise reduction, both objectively and subjectively.

## 2. Materials and Methods

**Figure 2:**
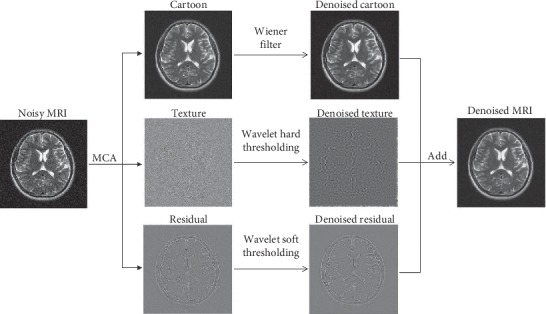
The flow chart of denoising an MR image.

In this study, we present a hybrid MR image denoising method based on MCA, Wiener filtering, and wavelet hard and soft thresholds. The whole denoising flow chart is shown in Figure 2. Firstly, add Gaussian white noise to the three original MR brain images. Secondly, use MCA to decompose the noisy images into three parts: cartoon, texture, and residual parts. In general, the residual part is regarded as a “noise component” and discarded directly by traditional MCA. Nevertheless, this study retains the residual part, which contains the outline of the brain. Thirdly, use Wiener filter, wavelet hard threshold, and wavelet soft threshold to remove noise in cartoon, texture, and residual parts, respectively. At last, superimpose the three denoised subimages to get denoised MR images.

### 2.1. Materials

The original data are a very thorough set of sequences of MR brain imaging of Axial T2, which was obtained from the open-edit educational radiology resource radiopaedia.org [19]. This paper chooses three static images at different moments of the original data for research. They are noise-free and of high resolution.

### 2.2. Wiener Filter

The Wiener filter is an optimal linear filter proposed by Norbert Wiener in 1942. It seeks the linear time-invariant filter whose output comes as close as possible to the original signal. In other words, the goal is to minimize the mean square error (MSE) between the expected noise-free signal and the actual output signal. The Wiener filter assumes that the input is the sum of valuable signals and noise, both of which are generalized stationary processes, and their second-order statistical characteristics are known. Therefore, it is not adaptive and always implemented in the frequency domain.

Formally, let *f*(*x*, *y*) be the input image and *g*(*x*, *y*) be the degraded image with some point-spread function *h*(*x*, *y*) and additive noise *η*(*x*, *y*). So, in the spatial domain, the blurred image is

(1) $$\begin{array}{c} g\left(x,y\right)=H{\left(x,y\right)}^{*}f\left(x,y\right)+\eta \left(x,y\right), \end{array}$$

where ^*∗*^ means two-dimensional convolution, *H*(*x*, *y*) is the blurring function, and additive noise *η*(*x*, *y*) often refers to Gauss white noise, uniform noise, etc. The Wiener filter treats images and noises as random processes, and the objective is to find an estimate $\hat{f}$ of the original image *f*(*x*, *y*)such that the MSE is minimum. The optimization problem is as follows:

(2) $$\begin{array}{c} \min\;e^{2}=E\left\{{\left(f-\hat{f}\right)}^{2}\right\}, \end{array}$$

where *E* means mathematical expectation. In the frequency domain, the optimization solution is given by [20]

(3) $$\begin{array}{c} \hat{F}\left(u,v\right)=\frac{H^{*}\left(u,v\right)}{\left({\left|H\left(u,v\right)\right|}^{2}+S_{\eta }\left(u,v\right)/S_{f}\left(u,v\right)\right)}, \end{array}$$

where *H*^*∗*^(*u*, *v*) is the complex conjugate of *H*(*u*, *v*), *S*_*η*_(*u*, *v*) is the power spectrum of the noise, and *S*_*f*_(*u*, *v*) is the power spectrum of the original image. If (*S*_*η*_(*u*, *v*)/*S*_*f*_(*u*, *v*)) is larger, the Wiener filter is smaller, so the frequency will be ignored. For more detailed information on the Wiener filter denoising, please refer to the textbook [21].

### 2.3. Wavelet Transformation

Wavelet denoising is widely used to remove noise from various signals, including one-dimensional signals (such as EEG) and two-dimensional signals (such as MR images). The algorithm is relatively simple to implement and has been proven effective in image denoising [22]. The energy obtained by using the wavelet transform usually concentrates on large coefficients, which correspond to the chief portion of the original signal because common noises such as Gaussian white noise do not correlate with wavelets. Therefore, wavelet coefficients with large amplitudes are mostly the required signals, while wavelet coefficients with small amplitudes are usually noise. Due to this property, the most common technique for reducing image noise by wavelet is thresholding. In this study, we use hard and soft thresholds.

The hard threshold is defined as follows:

(4) $$\begin{array}{c} {\hat{w}}_{j,k}=\left\{\begin{array}{cc} w_{j,k}, & \left|w_{j,k}\right|\geq \lambda ,\\ 0, & \left|w_{j,k}\right|<\lambda . \end{array}\right.. \end{array}$$

Moreover, the soft threshold is defined as follows:

(5) $$\begin{array}{c} {\hat{w}}_{j,k}=\left\{\begin{array}{cc} \mathrm{sgn}\left(w_{j,k}\right)\left(\left|w_{j,k}|-\lambda \right.\right), & \left|w_{j,k}\right|\geq \lambda ,\\ 0, & \left|w_{j,k}\right|<\lambda . \end{array}\right.. \end{array}$$

In both definitions, *w*_*j*,*k*_. is the wavelet coefficient and *λ* is the threshold value. Then, we can use ${\hat{w}}_{j,k}$ to perform the inverse wavelet transform to obtain the denoised image. Hard thresholding and soft thresholding are widely used and effective. It should be noticed that the hard threshold function is discontinuous when *w*_*j*,*k*_=±*λ*, which would cause oscillation in the denoised signal. On the contrary, the soft threshold method is continuous globally, but there is a constant error between *w*_*j*,*k*_ and ${\hat{w}}_{j,k}$ when *w*_*j*,*k*_ ≥ *λ*, which would reduce the accuracy of the approximation. Therefore, the choice of threshold is the primary concern for the wavelet transform denoising and should be validated by experiments. More detailed theories of the wavelet transform with its application could be found in many pieces of the literature, such as in [23].

### 2.4. Our Proposed Method Based on MCA

The main idea of MCA is to decompose an image into different additive layers, and each layer corresponds to a kind of morphological component of the image. Besides, the layer decomposition is required to optimize the sparsity of its representation. The core method of layer decomposition is to use adapted dictionaries, one for texture part representation and the other for cartoon part representation. The dictionaries are mutually unrelated. Each dictionary can only sparsely represent one morphological component and cannot sparsely represent other morphological components. The algorithm has been proven to perform well in many applications.

Formally, let *s* be a signal, which could be divided into *K* parts. Let

(6) $$\begin{array}{c} s=\overset{K}{\underset{k=1}{\sum }}s_{k}, \end{array}$$

where each *s*_*k*_ represents a different type of signal decomposed from the signal *s*. For each possible representation *s*_*k*_, there must be a dictionary Φ_*k*_ ∈ *M*^*N*×*L*_*k*_^ (where *L*_*k*_ ≫ *N* normally) such that the optimization problem

(7) $$\begin{array}{c} \begin{array}{cc} {\bar{\alpha }}_{k}=\underset{\alpha }{\arg\min}{\left\|\alpha \right\|}_{0}, & \text{subject }\mathrm{to}\;s_{k}=\Phi_{k}\alpha , \end{array} \end{array}$$

Has a very sparse solution ($\left.{\left\|{\bar{\alpha }}_{k}\right\|}_{0}\right.$is very small). On the contrary, the optimization problem

(8) $$\begin{array}{c} \begin{array}{cc} {\bar{\alpha }}_{l}=\underset{\alpha }{\arg\min}{\left\|\alpha \right\|}_{0}, & \text{subject }\mathrm{to}\;s_{l}=\Phi_{k}\alpha \;\left(k\neq l\right), \end{array} \end{array}$$

Does not have a sparse solution.

For the decomposition of a signal, the MCA requires to optimize the following equation:

(9) $$\begin{array}{c} \begin{array}{cc} \left\{{\bar{\alpha }}_{1},{\bar{\alpha }}_{2},\ldots ,{\bar{\alpha }}_{K}\right\}\underset{\left\{\alpha_{1},\alpha_{2},\ldots ,\alpha_{K}\right\}}{\arg\min}\overset{K}{\underset{k=1}{\sum }}{\left\|\alpha_{k}\right\|}_{0}, & \text{subject }\mathrm{to}\;s=\overset{K}{\underset{k=1}{\sum }}\Phi_{k}\alpha_{k}. \end{array} \end{array}$$

For the decomposition of an image, the MCA usually decomposes it into three components: cartoon, texture, and additive noise. Then, we can throw away the noise component and add only cartoon and texture components as the denoised image.

In this study, MCA separated two morphological components with different features and different dictionaries from the MR image, namely, the cartoon and texture parts. Accordingly, the optimization equation is as follows: for an MR image *s*,

(10) $$\begin{array}{c} \begin{array}{cc} \left\{{\bar{\alpha }}_{c},{\bar{\alpha }}_{t}\right\}=\underset{\left\{\alpha_{c},\alpha_{t}\right\}}{\arg\min}\left({\left\|\alpha_{c}\right\|}_{0}+{\left\|\alpha_{t}\right\|}_{0}\right), & \text{subject }\mathrm{to}\;s=\Phi_{c}\alpha_{c}+\Phi_{t}\alpha_{t}, \end{array} \end{array}$$

where Φ_*c*_ and Φ_*t*_ represent the overcomplete dictionaries of cartoon and texture parts, respectively. *α*_*c*_ and *α*_*t*_ are the corresponding sparse coefficients. Because this is an NP-hard problem, *l*_1_-norm would be used for approximation [24]. On the contrary, a noisy MR image cannot be accurately decomposed into cartoon and texture parts of sparse representations. So, a less strict constraint can be used to approximate the decomposition. The optimization equation used is as follows:

(11) $$\begin{array}{c} \left\{{\bar{\alpha }}_{c},{\bar{\alpha }}_{t}\right\}\underset{\left\{\alpha_{c},\alpha_{t}\right\}}{\arg\min}\left({\left\|\alpha_{c}\right\|}_{1}+{\left\|\alpha_{t}\right\|}_{1}\right),\text{subject }\mathrm{to}\;{\left\|s-\Phi_{c}\alpha_{c}-\Phi_{t}\alpha_{t}\right\|}_{2}\leq \varepsilon , \end{array}$$

where *ε* is the value of noise tolerance. The solution of the problem results in that the decomposition would leave out some components (i.e., the residual), which cannot be sparsely represented by both dictionaries. Let *R* be the residual part. Then, our decomposition model can be summarized as follows:

(12) $$\begin{array}{c} s=\Phi_{c}\alpha_{c}+\Phi_{t}\alpha_{t}+R. \end{array}$$

As shown in Figure 1, the MCA decomposes the original MR image into the cartoon, texture, and residual parts, which represent a meaningful component, an insignificant component, and the residual part, respectively.

After the noise-added MR image is decomposed into three components, each component is denoised by different methods. Specifically, the Wiener filter, wavelet hard threshold, and wavelet soft threshold are used to denoise the cartoon, texture, and residual parts, respectively. Finally, all the denoised subimages are superimposed together as the final denoised MR image. The flow chart of denoising an MR image is shown in Figure 2.

### 2.5. Evaluation Methods

There are two ways to evaluate the performance of different image denoising methods: objective method and subjective method. The subjective evaluation method is to compare the original image and the denoised image visually with naked eyes. The objective evaluation method is an index to quantify denoising performance. Here, we employ two common objective evaluation indexes: mean square error (MSE) and peak signal-to-noise ratio (PSNR).

The mean square error (MSE) is calculated as follows:

(13) $$\begin{array}{c} \text{MSE}=\frac{1}{M\times N}{\overset{M-1}{\underset{i=0}{\sum }}\overset{N-1}{\underset{j=0}{\sum }}\left(f_{ij}-{\hat{f}}_{ij}\right)}^{2}, \end{array}$$

where *M* and N represent the length and width of the image, respectively, *f*_*ij*_ denotes the pixel value of the original image, and ${\hat{f}}_{ij}$ represents the pixel value of the denoised image.

The peak signal-to-noise ratio (PSNR) is computed as follows:

(14) $$\begin{array}{c} \text{PSNR}=10\;*\;\log\frac{L^{2}}{\text{MSE}}, \end{array}$$

where *L* represents the maximum grayscale value of the pixels in an image. Here, *L* = 255.

## 3. Analysis of Results

In this section, all of the simulation experiments were accomplished in Matlab 2012. The Wiener filter, wavelet hard threshold, wavelet soft threshold, and proposed method (a combined method of the previous three methods based on MCA) are adopted for experimental comparison.

### 3.1. Objective Evaluation

In this section, the noise reduction effects are achieved when the mean value of Gaussian noise is zero, and the noise variance is 0.01, 0.03, 0.05, 0.07, and 0.09, respectively.

**Figure 3:**
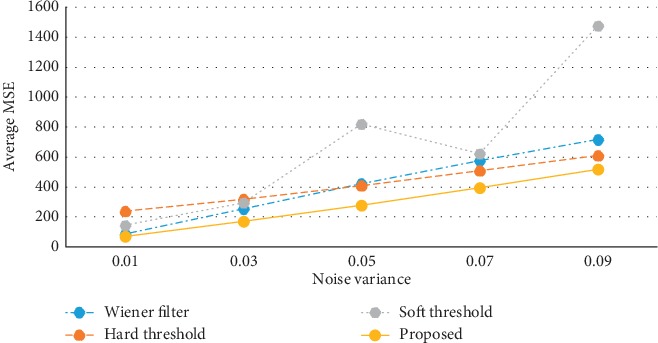
Average MSE values of the three MRI images in different ways.

**Figure 4:**
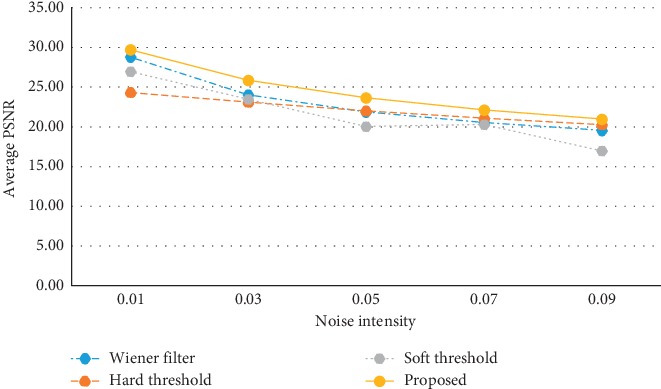
Average PSNR values of the three MRI images in different ways.

The objective comparison results of the three MRI images are shown in Tables 1–3, respectively. The tabulation results indicate that the MSE values of the proposed method are always significantly lower than other methods, while the PSNR values of that are always higher than other methods. More intuitively, the line graphs in Figure 3 and Figure 4 visualize the average MSE values and the average PSNR values of the three MRI images, respectively. It is observed that the average MSE line of the proposed method is lower than other methods, and the average PSNR line of the proposed method is higher than other methods. To sum up, the proposed method achieves better denoising effects in terms of the MSE value and the PSNR value than each method alone.

### 3.2. Subjective Evaluation

In this section, the denoising effects are achieved when the mean value of Gaussian noise is zero and the variance of that is 0.05.

**Figure 5:**
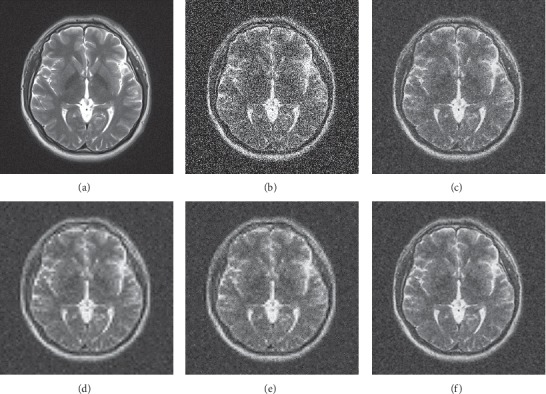
Subjective denoising results of MRI 1 in different ways. (a) Original image, (b) noisy image, (c) Wiener filter, (d) hard threshold, (e) soft threshold, and (f) proposed.

**Figure 6:**
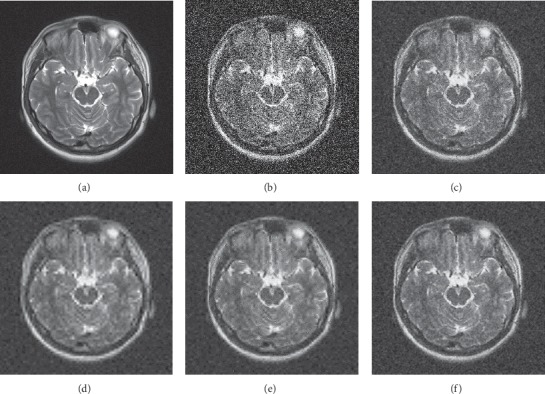
Subjective denoising results of MRI 2 in different ways. (a) Original image, (b) noisy image, (c) Wiener filter, (d) hard threshold, (e) soft threshold, and (f) proposed.

**Figure 7:**
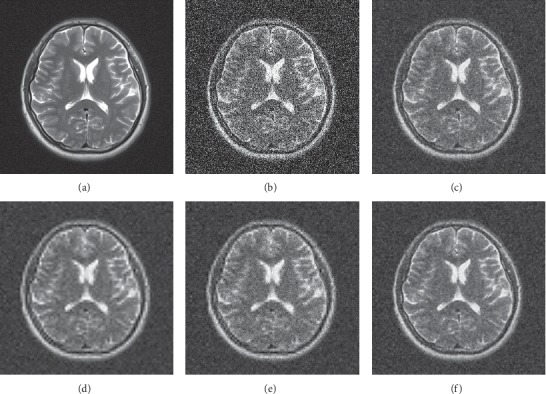
Subjective denoising results of MRI 3 in different ways. (a) Original image, (b) noisy image, (c) Wiener filter, (d) hard threshold, (e) soft threshold, and (f) proposed.

The denoising visual effects of the three MRI images are shown in Figures 5–7. The images denoised by hard and soft thresholds lose more important outline information than the other methods and therefore appear blurry. On the contrary, the images denoised by the Wiener filter retain most of the edge information as well as considerable noises. By contrast, the images denoised by the proposed method preserve more edge information and less noise.

## 4. Discussion

The proposed method achieves better denoising effects than other concerned methods, both objectively and subjectively. Let us explore the relative optimality of the proposed method.

Our goal is to use a combination of classical spatial and transform domain filters to achieve better denoising effects. The MCA can decompose an image into different morphological components, which enables us to combine different methods conveniently. The Wiener filter is a spatial domain filter that can preserve most of the edge information. The wavelet thresholding is a transform domain filter based on the property of sparsity. It can amplify the dissimilarity between noise and true signal by transformation and then use thresholding functions to reduce noise. There are two common thresholding functions: hard thresholding and soft thresholding. Hard thresholding gives better results by preserving edge information in some cases [25]. Soft thresholding tends to over smoothen the restored image. Transform domain methods can represent textures and low contrast information [3].

Decompose the noise-added MR images by MCA into three parts: the cartoon, texture, and residual parts. The denoising methods used in the proposed method are the Wiener Filter, hard threshold, and soft threshold. As an experiment validation, we will use all possible combinations of the three methods to remove noises in the three parts. To keep things simple, denote by *W*, *H*, and *S* the Wiener Filter, hard threshold, and soft threshold, respectively.  Table 4 shows the PSNR values of different methods used in the three parts of the images. For example, WHS means using the Wiener filter, hard threshold, and soft threshold to remove noises in the cartoon, texture, and residual parts, respectively. In contrary, WHS is our proposed method. It is observed that the proposed method always has the highest PSNR values except when the noise variance in MRI 1-2 is 0.01. However, the average PSNR values of the proposed method still reach the highest even when the noise variance is 0.01.  Table 5 reveals the fact.

As an exploratory research method, the proposed MRI denoising method is relatively effective, but the denoising effect is not so satisfactory. In the future, we will study more excellent denoising methods for MRI and fMRI.

## 5. Conclusion

In summary, we can illustrate our work in three steps. Firstly, describe the merits and drawbacks of traditional image denoising methods: Wiener filter, wavelet hard threshold, and wavelet soft threshold. Secondly, propose a comprehensive denoising algorithm based on the morphological component analysis. It can be briefly described as follows. Separate a noise-added image into three parts: two components that can be sparsely represented (cartoon and texture parts) and one residual part that cannot be sparsely represented (the residual part). Then, use the Wiener filter, wavelet hard threshold, and wavelet soft threshold to denoise the cartoon, texture, and residual parts, respectively. Finally, reconstruct the denoised image by adding the three denoised parts. Thirdly, analyze the relative best performance of the proposed method objectively and subjectively, explain our original intention, and verify the experimental results.

## Figures and Tables

**Table 1:** Denoising results of MRI 1 in different ways.

Results	MSE of MRI 1					PSNR of MRI 1				
Noise variance	0.01	0.03	0.05	0.07	0.09	0.01	0.03	0.05	0.07	0.09
Wiener filter	87	257	422	577	723	28.75	24.03	21.88	20.52	19.54
Hard threshold	252	334	425	523	628	24.04	22.89	21.85	20.95	20.15
Soft threshold	74	231	382	702	1043	29.44	24.49	22.31	19.67	17.94
Proposed	73	174	284	399	522	29.48	25.73	23.6	22.13	20.95

**Table 2:** Denoising results of MRI 2 in different ways.

Results	MSE of MRI 2					PSNR of MRI 2				
Noise variance	0.01	0.03	0.05	0.07	0.09	0.01	0.03	0.05	0.07	0.09
Wiener filter	87	254	417	566	701	28.76	24.09	21.93	20.6	19.67
Hard threshold	233	308	397	493	586	24.46	23.25	22.14	21.2	20.45
Soft threshold	132	319	1682	436	2591	26.91	23.09	15.75	21.73	13.88
Proposed	68	167	272	388	501	29.79	25.92	23.78	22.24	21.13

**Table 3:** Denoising results of MRI 3 in different ways.

Results	MSE of MRI 3					PSNR of MRI 3				
Noise variance	0.01	0.03	0.05	0.07	0.09	0.01	0.03	0.05	0.07	0.09
Wiener filter	85	256	423	583	730	28.85	24.05	21.87	20.48	19.5
Hard threshold	232	311	404	510	618	24.45	23.2	22.07	21.06	20.22
Soft threshold	230	339	393	728	797	24.5	22.81	22.19	19.5	19.11
Proposed	68	167	278	400	530	29.85	25.9	23.7	22.11	20.89

**Table 4:** PSNR values of denoising the cartoon, texture, and residual parts in order (*W*: Wiener filter, *H*: hard threshold, and *S*: soft threshold).

Noise variance	0.01	0.03	0.05	0.07	0.09
PSNR of MRI 1					
SHW	29.34	25.5	23.52	21.42	20.99
SWH	28.7	24.07	22	20.06	19.67
HSW	24.7	23.02	19.49	19.65	17.66
HWS	24.34	22.24	20.91	19.83	19.13
WSH	29.77	25.32	20.25	20.34	18.03
WHS (proposed)	29.48	25.73	23.6	22.13	20.95
Max	29.77	25.73	23.6	22.13	20.99

PSNR of MRI 2					
SHW	29.24	23.99	23.63	22.14	21.16
SWH	28.19	22.72	21.94	20.62	19.75
HSW	24.98	23.32	15.52	20.54	13.89
HWS	24.47	22.34	21.03	20.04	19.32
WSH	29.81	25.56	15.78	21.27	14.01
WHS (proposed)	29.79	25.92	23.78	22.24	21.13
Max	29.81	25.92	23.78	22.24	21.16

PSNR of MRI 3					
SHW	29.51	25.66	23.26	21.46	20.81
SWH	28.73	24	21.86	20.08	19.6
HSW	22.48	22.95	20.65	19.69	18.57
HWS	24.7	22.41	20.95	19.88	19.18
WSH	24.49	24.91	21.54	20.3	18.95
WHS (proposed)	29.85	25.9	23.7	22.11	20.89
Max	29.85	25.9	23.7	22.11	20.89

**Table 5:** Average PSNR values of denoising the cartoon, texture, and residual parts in order (*W*: Wiener filter, *H*: hard threshold, and *S*: soft threshold).

Noise variance	0.01	0.03	0.05	0.07	0.09
SHW	29.36	25.05	23.47	21.67	20.99
SWH	28.54	23.60	21.93	20.25	19.67
HSW	24.05	23.10	18.55	19.96	16.71
HWS	24.50	22.33	20.96	19.92	19.21
WSH	28.02	25.26	19.19	20.64	17.00
WHS (proposed)	29.71	25.85	23.69	22.16	20.99
Max	29.71	25.85	23.69	22.16	20.99

## Data Availability

The data used to support the findings of this study are available from the corresponding author upon request.
